# Chromium Concentrate Recovery from Solid Tannery Waste in a Thermal Process

**DOI:** 10.3390/ma13071533

**Published:** 2020-03-27

**Authors:** Stanisław Famielec

**Affiliations:** Faculty of Production and Power Engineering, University of Agriculture in Krakow, 30-149 Kraków, Poland; stanislaw.famielec@urk.edu.pl; Tel.: +48-12-662-46-60

**Keywords:** tannery waste, chromium recovery, chromium concentrate, waste incineration

## Abstract

Leather processing requires substantial inputs of energy, water and chemicals. Additionally, it generates significant amounts of liquid and solid waste, severely impacting the environment. Processing 1 Mg of raw hides yields up to 600–700 kg of waste, considerable amounts of which are solid tannery waste. Such waste contains chromium (Cr) compounds, which are commonly used as tanning agents. This paper reviews solid tannery waste treatment technologies, with emphasis on waste incineration in a specially designed experimental tunnel incinerator. Three different types of tannery waste were subjected to tests: trimmings, shavings and buffing dust. As the research revealed, the process can be applied to all types of solid tannery waste. Moreover, it enables the reuse of the heat of the process and results in a Cr concentrate in the process residues. The conducted analyses (carbon, hydrogen and nitrogen elemental analysis; inductively coupled plasma optical emission spectroscopy; powder X-ray diffraction) proved that there is no or little organic content in the obtained residual ash, which contains up to 53.1%(w/w) Cr in the form of Cr (III) oxide. Such material may be used as a Cr ore substitute in the chemical or metallurgical industries.

## 1. Introduction

The tanning industry is considered one of the most environmentally unfriendly industrial activities [1]. Tanning, namely processing raw hides into a useful product called leather, requires not only a considerable material and energetic input (process water, chemicals, process heat) but also generates significant amounts of wastewater, air pollution and solid waste as an output [2,3]. Tanning is a multi-stage process, consisting of pre-treatment operations, the so-called “proper” tanning (stabilization of the hide collagen structure with tanning agent molecules) and finishing operations [4].

Out of 1 Mg of raw hides, approx. 200–250 kg is converted into finished leather, and the rest becomes waste during the subsequent process stages [5]. There are different types of solid tannery waste, depending on the process stage during which the waste is generated. They can be divided into three main groups [5,6,7]:

1. Untanned solid tannery waste (USTW), such as trimmings (unused pieces of hides) or fleshings (non-leather tissues that have been mechanically scraped off). This waste is prone to biologically-mediated decay.
2. Tanned solid tannery waste (TSTW), generated in the operations conducted during or after the proper tanning process, i.e., leather trimmings, splits, shavings and dust. These types of waste are as biologically stable as the leather itself, as they contain the same tanning agent compounds in their inner structure.
3. Tannery sludge (TS) obtained as a result of process wastewater treatment. TS contains organic matter leached from hides/leather in tanning operations, as well as excess chemicals used throughout the whole tanning process.

**Figure 1:**
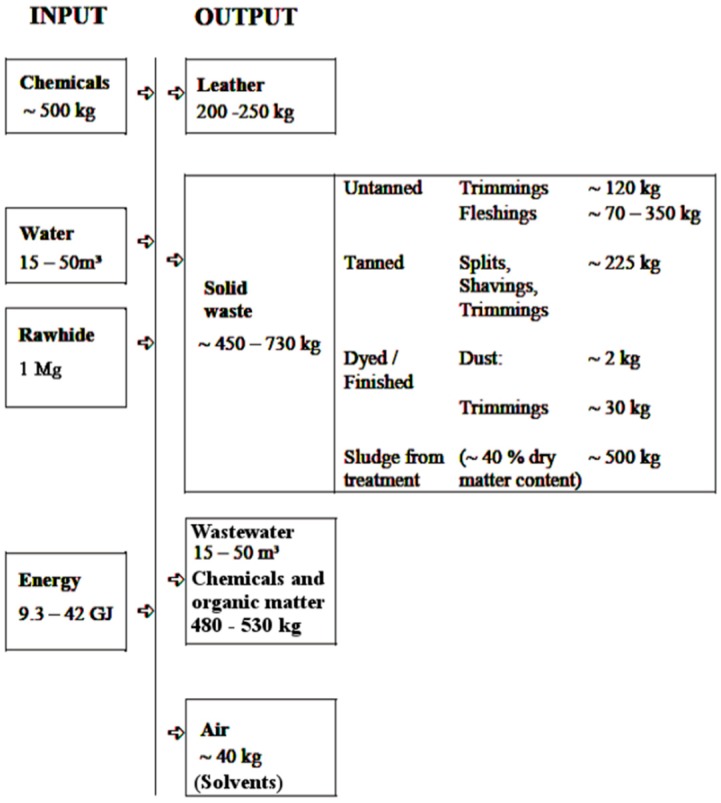
Input/output overview of a conventional chrome-tanning process for bovine salted hides per 1 Mg of raw hide processed [5].

The amounts of solid waste generated during a typical tanning process is showed in Figure 1.

In 90% tanneries worldwide, chromium (Cr) (III) salts are used as tanning agents [8,9]. During the tanning process, Cr compounds cross-link collagen fibers. In this process a significant excess of a tanning agent is applied. The unused Cr compounds remain in wastewater and eventually constitute TS [2]. As a result of tanning, the processed material contains up to 3%–4%(w/w) Cr [1]. Such Cr content is typical not only for the final product (leather), but also for all types of TSTW. The presence of Cr is one of the major problems related to tannery waste treatment—it endangers the environment and human health [3,10]. Cr is considered the second most abundant inorganic ground-water contaminant at hazardous waste sites [11]. While Cr(III) in most cases reacts to form insoluble compounds and is commonly considered as nontoxic, in certain conditions (e.g., in the presence of other redox-active metals, such as manganese), oxidation to Cr(VI) may take place [12]. Hexavalent Cr is recognized by the International Agency for Research on Cancer and by the US Toxicology Program as a pulmonary carcinogen [13]. If untreated, the tannery waste may cause a serious threat to the environment, especially to soil and groundwater. It has already been stated that in some areas, the tannery effluents and waste contribute to changing the chemistry of soil solution, which results in the introduction of toxic metals (Cr, Pb and others) into the soil environment [14].

According to the latest world statistical compendium on raw hides and skins issued by Food and Agriculture Organization of the United Nations (FAO) [15], the worldwide annual production of bovine hides and skins amounts to approx. 6.5 million Mg (wet salted weight), of which more than 4.3 million Mg is processed in developing countries. Sheepskins, lambskins, goatskins and kidskins are processed globally for approx. 750 thousand Mg (dry weight). Therefore, by rough calculation, approx. 3.5–4.0 million Mg of solid waste is generated worldwide annually from the tanning industry, of which a significant share (up to 30%–35% [5]) may contain Cr.

Treatment of tannery waste has always been a serious issue for leather manufacturers. Tannery waste is produced in huge amounts, different types, with complex chemical content (lime, salts, acids, Cr and other metals) and high water content. All these factors contribute to the fact that waste management in the tanning industry in many cases fails to meet Waste Hierarchy and environmental protection standards [2,3]. Conventionally, USTW were used as fertilizers, in hide-glue and gelatin production or as a source of nutrients, whereas TSTW were utilized to some extent as a material in production of leather fiberboards or so-called “secondary” leather, which is a composite material similar to leather, appropriate for certain applications in footwear manufacturing [3,16]. In developing and under-developed countries, landfilling predominates as a method for solid tannery waste treatment [17,18]. Tannery waste landfilling was also common in certain EU member states, e.g., in Poland [3] and the Czech Republic [10]. However, due to global trends towards a circular economy and the pursuit of new resources, tannery wastes have recently been identified as “new raw materials” [19], with a high potential for recovery of energy, organics or valuable elements (e.g., Cr).

Some waste types, especially USTW, can be subjected to enzymatic hydrolysis or collagen extraction processes, which allows the recovery of valuable materials, such as feed additives [20], biodegradable packaging materials [21,22] or collagen-based biomaterials [23]. In the case of TSTW, the presence of Cr hinders such applications. However, Cr removal can be conducted during an operation known as “reverse tanning”, which technically consists of a few extraction processes with different solutions. Cr compounds obtained via this method can be regenerated to a tanning agent to be used in further tanning operations [22,24]. Once Cr is removed, TSTW may undergo standard or enzymatic hydrolysis processes, similar to USTW [10]. Agustini et al. [18] and Priebe et al. [25] investigated the potential of TSTW and TS as a feedstock in biogas production. Those waste types appeared to be anaerobically biodegradable [18]; however, the increase of Cr concentration in the substrate significantly reduced the biogas and methane yield [25]. Several authors indicate that TSTW, especially shavings, are characterized by high adsorption capacity—Piccin et al. [26] removed organic dyes from tannery effluents at high efficiencies by adding shavings, whereas Chabaane et al. [27] and Oliveira et al. [28] tested shavings as an adsorbent of toxic Cr(VI) or As(V) from aqueous media.

Among the different solid waste treatment options available, thermal treatment methods are considered suitable for TSTW [1,4]. They include incineration, gasification and pyrolysis, and are applied mainly in order to reduce waste volume as well as for production of energy (incineration) or combustible gaseous and/or liquid products (gasification, pyrolysis) [16]. Organic substances predominate in tanned waste dry matter (total organic matter up to 87.5% in dry basis [6]) and the energy content of TSTW is relatively high (exceeding 15–16 MJ·kg^−1^) [6,29], which indicates that such waste can be successfully used as fuel. However, the presence of some elements in this waste (Cr, nitrogen, sulfur) requires additional precautions to ensure environmental standards are met during thermal treatment [30]. Subjecting chrome-tanned waste to high temperatures in the presence of oxygen (or under oxidative atmosphere of other type) may lead to the oxidation of Cr(III) to carcinogenic Cr(VI) [10,16]. Nitrogen atoms bound in the collagen structure of TSTW during thermal processes react to form gaseous products, such as NH_3_ and HNCO (pyrolysis) or NO_x_ (combustion) [31]. Further on, high water content in some groups of waste (e.g., in fresh shavings) influence the process negatively and an additional stage (waste drying) may be required [3]. All the above-mentioned aspects have to be taken into consideration in developing new technologies for thermal treatment of tannery waste. 

As a result of subjecting TSTW to pyrolysis it is possible to obtain carboneous residue (chars) which is suitable as solid fuel (calorific value up to 25 MJ·kg^−1^) or as activated carbon, with a surface area reaching 800 m^2^·g^−1^ [24]. Velusamy et al. [16] reported up to a 52% yield of bio-oil for commercial fuel production as a product of TSTW pyrolysis. A double pyrolysis process can be applied in order to reduce the amount of solid residue and increase the share of gaseous products [32]. Kluska et al. [29,30] investigated the process of combustion and co-combustion of pelletized leather tannery waste and hardwood pellets indicating that while there are similarities of both fuels in terms of combustion velocity and obtained maximum temperatures, the emission of nitrogen oxides and amounts of process residues (ash) is much higher for pelletized tannery waste. The ash from TSTW incineration contains a significant share of Cr(III) oxide (Cr_2_O_3_) [30,33]; however, the presence of Cr(VI) in the ash was also reported [16]. As many authors indicate, Cr oxidation from Cr(III) to Cr(VI) depends on the presence of other substances as well as on process conditions [34,35,36]. The tunnel system for tannery waste incineration used by Famielec [37] enables the control of the thermal conditions and the maintenance of relatively low temperatures in the combustion zone (slightly more than 850 °C), which hinders the oxidation of Cr(III). However, fly ash, NO_x_ and SO_2_ emissions exceeding the emission standards were noticed in this experiment, indicating that TSTW incineration systems require a separate process for proper flue gas treatment.

Cr-enriched products of tannery waste thermal treatment could be a valuable source of Cr for industry and a substitute for chromite ore, which is an essential raw material for the production of ferrochrome, the substrate for stainless steel manufacturing. Cr is the main element providing corrosion resistance in stainless steels and is also added to a special grade of alloy steels used in the manufacture of tools [38]. Chromite ore mine production amounted to approx. 43 million Mg in 2018 worldwide [39]. More than 90% of mined chromite is consumed by the metallurgical industry, and the rest is used in the chemical and foundry sand industry [40]. Currently, with the depletion of high-grade lumpy chromite ores, the efficient use of low-grade chromite fines and Cr concentrates becomes increasingly important in the aspects of economic efficiency and sustainable utilization of remaining chromite resources [41,42]. Cr recovery in the tanning industry has been carried out mainly in order to remove Cr from tannery effluents [43,44]; however, there are several studies focused on obtaining Cr-enriched products from TSTW. El-Sheikh and Rabbah [45], using tannery waste as substrate, synthesized nano-crystalline magnesium chromites for application as high-temperature ceramics, strengthening agents or interconnect materials for solid oxide fuel cells. Wang et al. [46] applied leaching and ion exchange to efficiently remove Cr (III) from TSTW. The ash from tannery waste incineration was reported to be suitable as a substrate substitute for sodium chromate production [1,6,16]. Alves et al. [47] investigated the possibility of recycling Cr contained in ashes from leather shaving incineration and indicated its utility as a raw material for the production of commercial high-carbon ferrochrome alloy (HC-FeCr) by carbothermal reduction at a temperature of 1600 °C. The ash used in this research contained approx. 40%–50% Cr_2_O_3_.

The aim of this study was to investigate the possibility of applying the process of TSTW incineration conducted in a tunnel incinerator in order to obtain ash with a high content of Cr (as Cr_2_O_3_) for further potential applications.

## 2. Materials and Methods 

### 2.1. Materials

TSTW for experiments (shavings, trimmings, buffing dust) were collected at a tannery in southern Poland. All the waste types came from the same production line for bovine hides processing, therefore there were no substantial differences in their chemical composition. They differed mostly in moisture content (MC) and in size and shape of individual waste particles. Elemental composition of the waste, presented in Table 1, was determined using a CHNSO Flash 2000 analyzer (Thermo Scientific, Waltham, MA, USA) and an energy dispersive X-ray fluorescence spectrometer MiniPal 4 (PANalytical, Almelo, The Netherlands).

Three samples of waste were prepared and tested in the research:

(1) tanned trimmings (lower heating value (LHV) approx. 17,900 kJ·kg^−1^; MC approx. 10%(w/w));
(2) shavings (LHV approx. 9100 kJ·kg^−1^; MC approx. 41%(w/w));
(3) mixture of leather trimmings, shavings and buffing dust, in a weight ratio of 2:2:1 (LHV approx. 14,100 kJ·kg^−1^; MC approx. 23%(w/w)).

MC was determined by oven drying of material samples at 105 °C until constant weight (according to PN-EN ISO 18134-3:2015-11), while LHV was measured using a C6000 isoperibol calorimeter (IKA, Staufen, Germany) according to PN-EN 14918:2010.

### 2.2. The Experimental Installation

**Figure 2:**
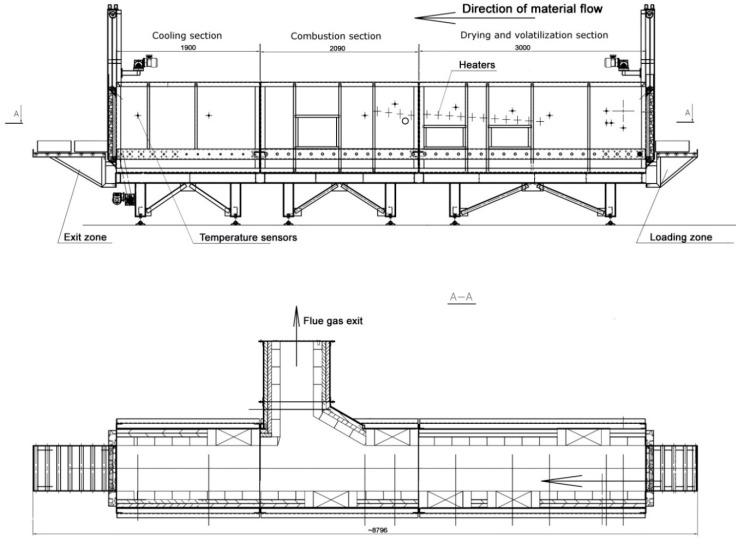
A scheme of the tannery waste incinerating installation.

The installation for waste incineration was specially designed and manufactured for experiments by Firma Czylok (Jastrzębie-Zdrój, Poland) and located in a tannery in southern Poland. The installation consists of a tunnel incinerator, heat exchangers, flue gas outlet system and a control panel. The system is designed to allow the maintenance of thermal conditions which continuously change along the length of the tunnel. Thus, it can be virtually divided in a few sections corresponding to the following processes: waste drying, volatilization of organic matter and combustion. The tunnel is approx. 7 m long. The waste is put in special steel containers with perforated bottoms (to allow air circulation), which are transmitted through the tunnel by a roller system. Preheating and maintaining the temperature during the process is possible due to three sections of electric heaters. The nominal power of the total system is 78 kW. The system is equipped with temperature (Ampero, Katowice, Poland), pressure (Aplisens, Warszawa, Poland) and oxygen sensors (Bosch, Abstatt, Germany), which give information about temperature at several points of the system, pressure at the entrance and in the combustion section, as well as oxygen concentration in the flue gas. The heat exchanger system enables cooling the flue gas by heating the process water used in the tannery and pre-heating the intake air for the incineration process. The flue gas eventually goes out through a chimney equipped with measurements inlets for flue gas analyzers. The scheme of the experimental installation is presented in Figure 2.

### 2.3. Process Conditions

Three tests (one for each sample) were conducted. In each test containers with waste (3 ± 0.1 kg per container) were introduced to the tunnel. The time interval between the introduction of containers was 12 min. This gives 5 containers (or 15 ± 0.5 kg of waste) per hour. The total time of container transmission through the tunnel was approx. 1 h. Since the roller speed was constant, the time of processing the waste though each section of the tunnel was related to the section’s length. The waste remained in the first section (drying and volatilization) for approx. 25 min, in the second section (combustion) for approx. 18 min and in the last section (cooling) for approx. 17 min. The temperature of the preheated combustion air was 350 °C. Before the beginning of the experiment, the tunnel was preheated to the temperature exceeding 700–750 °C. The temperature in the combustion section during experiments exceeded 850 °C—the minimum temperature of waste incineration allowed by EU regulations [48].

During the tests some electrical energy input was necessary to preheat the process air, to maintain temperature in each section and to run fans and the roller system. It amounted to approx. 123–165 MJ per test (lasting approx. 1 h), depending on the input material (more energy used in the case of shavings, less for tanned trimmings). Heat released from the waste in each test depended on LHV of the material and ranged from 136.8 MJ (shavings) to 268.5 MJ (tanned trimmings). The heat exchanger system allowed for recovery of approx. 130–135 MJ heat in an hour-long single test by heating the process water used in the tannery works for hide processing. The recovery rate could have been higher; however, due to technical limitations the temperature of the inlet water was relatively high (approx. 55 °C) and therefore less heat was utilized than expected. Other significant outgoing heat fluxes included chimney loss, losses through construction elements and heat accumulation in the incinerator lining.

### 2.4. Analyses

The residue ash was weighted using Radwag PS.6100.R2.M balance and subsequently analyzed using powder X-ray diffraction (XRD). XRD measurements were performed on an X’pert Phillips diffractometer (Almelo, The Netherlands) using a CuKa radiation (λ= 1.54178 Å) in 2Theta range from 10° to 90°. A Perkin Elmer 2400 CHN Elemental Analyzer (Boston, MA, USA) was used for carbon, hydrogen and nitrogen (CHN) elemental analysis of ash after combustion. Cr concentration in ash samples was analyzed using a Perkin Elmer OPTIMA 7300DV inductively coupled plasma optical emission spectroscopy (ICP-OES) analyzer (Shelton, CT, USA). A titration method (using reagent grade Mohr’s salt and potassium manganate (VII) purchased at POCH, Gliwice, Poland) was applied to check the presence of Cr (VI) content in the ash.

## 3. Results and Discussion

The amounts of ash obtained after combustion of the waste samples were: 1.00 ± 0.05 kg or 7.4%(w/w) for tanned trimmings, 0.93 ± 0.05 kg or 10.5%(w/w) for shavings and 1,06 ± 0.05 kg or 9.2%(w/w) for the mixture of three TSTW types. Table 2 presents the content of C, H, N and Cr in the ash.

The CHN analysis for the ash samples revealed that there was no hydrogen or nitrogen present (below detection limits which is 0.1%(w/w)) while the elemental carbon content amounted to 0.23–0.36%(w/w). It proves that the combustion process was practically complete and only a small amount of organic content was present in the product. Total Cr concentration in the ash ranged from 39.1%(w/w) (shavings) to 53.1%(w/w) (tanned trimmings), which indicated that Cr compounds predominate in the ash.

**Figure 3:**
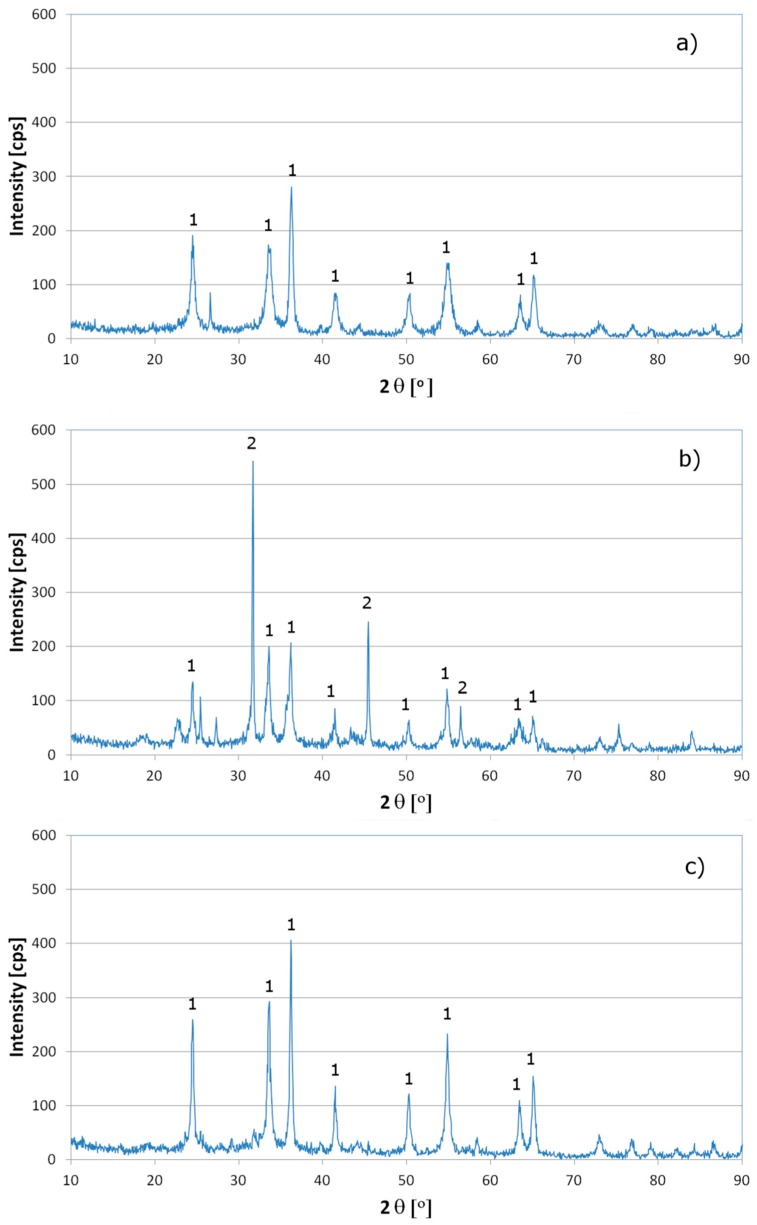
X-ray diffraction (XRD) powder diffraction patterns of tannery waste ash samples: (**a**) tanned trimmings; (**b**) shavings; (**c**) mixture of leather trimmings, shavings and buffing dust, in a weight ratio of 2:2:1 (1—Cr_2_O_3_; 2—NaCl).

XRD patterns of ash samples are presented in Figure 3. For tanned trimmings and TSTW mixture, the only crystalline phase observed is Cr_2_O_3_. In the case of shavings, apart from Cr_2_O_3_, only the NaCl phase is present, which presumably results from the fact that in the tannery works raw salted hides are stored close to the stands where leather is shaved and shavings are collected. In all ash samples, no visible crystalline phase of any Cr(VI) compound is present. Titration with Mohr’s salt carried out for the ash samples obtained gave the same results as for a blank sample, which indicated that Cr(VI), if present in the ash, did not exceed the concertation of approx. 0.5 mg Cr·g^−1^ (detection limit for this method). Taking into account the fact that only the Cr_2_O_3_ phase is recognizable in the XRD patterns and no Cr(VI) was detected, it is justified to conclude that practically all Cr present in the ash is in oxidation state +3, predominantly as Cr_2_O_3_. The occurrence of Cr in other oxidation states is highly improbable [49].

As several researchers stated [10,16], the oxidation of Cr(III) to Cr(VI) during the incineration of tannery waste is very probable and in certain conditions may lead to the transformation of whole Cr content into Cr(VI) compounds. Such a process was observed during the incineration of TS in oxic conditions in the range of temperature exceeding 500 °C, resulting in the formation of Cr(VI) salts, mostly CaCrO_4_ [35]. Ferreira et al. [50], who tested the leachability of Cr(VI) from the ash after combustion of tanned leather in an electric furnace, reported that Cr(VI) concentrations in the ash vary depending on the temperature of combustion and holding time of leather samples in the furnace, and in some conditions (holding time of approx. 100–200 min, temperature lower than 600 °C or higher than 800–900 °C) can reach 20,000–30,000 mg Cr(VI)·kg^−1^ ash. Still, the formation of Cr(VI) from Cr(III) is not fully explained [35]. Some reports indicate that this reaction is not only related to temperature range but also to other process conditions and especially to the presence of other salts [34,36]. Verbinnen et al. [34], who investigated mixtures of Cr_2_O_3_ with K, Na and Ca salts incinerated up to 1100 °C, described the extent of Cr(III) oxidation to Cr(VI) as a function of temperature and added salts. Thus, the presence of oxygen and increase of temperature are not the sole factors for Cr(III) oxidation. The conditions of incineration in the conducted research allowed for obtaining the ash, in which oxidation of Cr(III) to Cr(VI) was hindered. Therefore, the ash obtained in this research is less dangerous to the environment and humans than in the case of products from incineration processes described by Velusamy et al. [16], who reported the presence of Cr(VI) in the ash, or Kavouras et al. [35], who reported the oxidation ratio from 74% to 100% of Cr(VI)/Cr_total_, depending on temperature.

The share of Cr in the ash was calculated to Cr_2_O_3_ and presented in Table 2. It was the highest in the case of incineration of trimmings (77.6%(w/w)) and exceeded the results presented by Kluska et al. [29,30], who observed the Cr_2_O_3_ share in the ash after incineration of shavings pellets in the range 21.33%–67.1%(w/w). High concentrations of Cr_2_O_3_ in the ash, exceeding the level of 45% which characterizes high quality metallurgical grade ore [51], indicate that the product obtained in this research is suitable for production of HC-FeCr, according to the method described by Alves et al. [47]. Worth noticing is the fact, that the range of Cr_2_O_3_ content in the researched ash is higher than in the ash used by Alves et al. (40%–50%) [47].

According to Nafziger [52], for the production of ferrochrome, the Cr/Fe ratio of the starting material should be as high as possible. As Johnson et al. [53] reported, with an increase of 25% for Cr content in ferrochrome, total energy consumption of austenitic stainless steel production decreases by 2.7%. Low-grade chromite ore has to be treated to increase the Cr content prior to subjecting to the smelting process, e.g., by carbochlorination [54]. The addition of Cr-enriched substances, such as the ash obtained in the presented research, to lean and complex ores or to other Cr-containing substrates (such as flue dust from ferrochrome production [55]) could allow for greater resource efficiency and higher recovery rates [56]. The possibility to produce ferrochrome from TS roasted at 650 °C and mill scale waste in an aluminothermic process was investigated by Wcisło et al. [57] on a laboratory scale, with positive results. The obtained alloy comprised approx. 72%–76% Cr, 24%–28% iron and less than 0.16% phosphorus. In this experiment, the Cr_2_O_3_ content in the substrate derived from TS was in the range of 70%–80%, which indicates that the ash obtained from the trimmings incineration in the tunnel system could constitute an appropriate substrate in ferrochrome production from waste by aluminothermy. The technology of melting steel in an electric arc furnace using Cr-containing waste instead of ferrochrome was presented by Ślęzak et al. [58], who applied briquettes of dried TS to the furnace input, together with high silicon cast iron scraps and slag-forming reagents. As the authors [58] stated, this technology allows for utilizing other waste types containing Cr; however, the Cr recovery rate depends on carbon content in the input mixture, which may require coke addition in the case of substrates with high Cr and low carbon shares (such as the ash from TSTW incineration).

Cr recovery from tannery waste or effluents carried out through leaching [46] or dissolving in acidic or alkaline solutions [24,43,44] aims mainly at obtaining a solution containing dissolved Cr (Cr(III) or Cr(VI) ions) as a final product, which can be further applied, e.g., as tanning liquor in a tannery. Treatment of wastewaters from wet recovery processes is necessary and may require additional infrastructure and energy input. TSTW incineration is a dry recovery method with no wastewater generated and the obtained Cr concentrate in the form of ash is a ready-to-use substrate for metallurgical processing. The tunnel system for TSTW incineration allows for obtaining the ash characterized by high Cr_2_O_3_ and low Cr(VI) contents, as well as for partial heat recovery. In order to provide a more complete techno-economic description of this technology, gas and fine particles emission, with a focus on potential Cr presence in the flue gas, will have to be investigated in further research. The carbon dioxide emission rate for the conducted research can be calculated on the basis of the input material composition, with the results varying from 14.6 kg CO_2_·h^−1^ for shavings, through 19.1 kg CO_2_·h^−1^ for the mixture of three TSTW types, to 22.3 kg CO_2_·h^−1^ for trimmings. Carbon dioxide emission coefficients for 1 kg of the ash obtained in each test amount to: 15.7 kg CO_2_·kg^−1^ ash for shavings, 17.9 kg CO_2_·kg^−1^ ash for the mixture of three TSTW types and 22.3 kg CO_2_·kg^−1^ ash for trimmings.

## 4. Conclusions

The presented results demonstrate that the TSTW incineration processes in a tunnel incinerator system are practically complete and the obtained ash (amounting to approx. 7.4%–10.5%(w/w) of incinerated waste depending on waste type input) contains mostly Cr_2_O_3_ and slight amounts of organic content. No evidence of any Cr (VI) phase was observed. The conducted research proves that problematic and potentially hazardous waste can be used as a source of raw materials, replacing limited natural resources in such important industrial branches as metallurgy or chemical industry. Such an approach corresponds to the requirements of a circular economy, which strongly encourages utilizing waste as renewable materials. By applying appropriate incineration conditions in a tunnel incinerator, it is possible to produce Cr concentrate from solid chrome-tanned waste. Nevertheless, further research, especially which secures the environmental safety of the process in terms of gaseous emissions, the entrainment of fine particles and the potential Cr emission with flue gas, is necessary to develop full industrial technology maturity.

## Figures and Tables

**Table 1:** Elemental composition of tested waste (%(w/w) in dry basis).

C	43.6–48.2
O	20.8–23.0
N	13.8–15.1
H	5.8–6.2
S	1.0–2.0
Cr	2.6–3.9
Na ^1^	1.2–3.1
Si ^1^	0.5–1.9
Cl ^1^	0.4–2.1
Fe, Ca	0.5–1.0
Mg, K, Al	<0.5

^1^ Higher values for shavings, mostly due to impurities (sand, NaCl) resulting from storing conditions.

**Table 2:** Content of C, H, N and Cr in the ash (% (w/w) in dry basis).

Element	Trimmings	Shavings	Mixture of Trimmings, Shavings and Buffing Dust
C	0.23	0.29	0.36
H	<0.1	<0.1	<0.1
N	<0.1	<0.1	<0.1
Cr	53.1	39.1	44.3
Cr_2_O_3_^1^	77.6	57.1	64.7

^1^ From calculations.
